# Zinc, ferritin, magnesium and copper in a group of Egyptian children with attention deficit hyperactivity disorder

**DOI:** 10.1186/1824-7288-37-60

**Published:** 2011-12-29

**Authors:** Magdy M Mahmoud, Abdel-Azeem M El-Mazary, Reham M Maher, Manal M Saber

**Affiliations:** 1 Pediatric department, Minia university, Minia city, Minia, postcode 61111, Egypt; 2 Clinical-pathology department, Minia university, Minia city, Minia, postcode 61111, Egypt

**Keywords:** Attention deficit, hyperactivity, zinc, ferritin, magnesium, copper

## Abstract

**Background:**

Attention deficit hyperactivity disorder is a behavioral syndrome of childhood characterized by inattention, hyperactivity and impulsivity. There were many etiological theories showed dysfunction of some brain areas that are implicated in inhibition of responses and functions of the brain. Minerals like zinc, ferritin, magnesium and copper may play a role in the pathogenesis and therefore the treatment of this disorder.

**Objective:**

This study aimed to measure levels of zinc, ferritin, magnesium and copper in children with attention deficit hyperactivity disorder and comparing them to normal.

**Methods:**

This study included 58 children aged 5-15 years with attention deficit hyperactivity disorder attending Minia University Hospital from June 2008 to January 2010. They were classified into three sub-groups: sub-group I included 32 children with in-attentive type, sub-group II included 10 children with hyperactive type and sub-group III included 16 children with combined type according to the DSM-IV criteria of American Psychiatric Association, 2000. The control group included 25 apparently normal healthy children.

**Results:**

Zinc, ferritin and magnesium levels were significantly lower in children with attention deficit hyperactivity disorder than controls (p value 0.04, 0.03 and 0.02 respectively), while copper levels were not significantly different (p value 0.9). Children with inattentive type had significant lower levels of zinc and ferritin than controls (p value 0.001 and 0.01 respectively) with no significant difference between them as regards magnesium and copper levels (p value 0.4 and 0.6 respectively). Children with hyperactive type had significant lower levels of zinc, ferritin and magnesium than controls (p value 0.01, 0.02 and 0.02 respectively) with no significant difference between them as regards copper levels (p value 0.9). Children with combined type had significant lower levels of zinc and magnesium than controls (p value 0.001 and 0.004 respectively) with no significant difference between them as regards ferritin and copper levels (p value 0.7 and 0.6 respectively).

**Conclusions:**

Children with attention deficit hyperactivity disorder had lower levels of zinc, ferritin and magnesium than healthy children but had normal copper levels.

## Background

Attention deficit hyperactivity disorder (ADHD) is a behavioral syndrome of childhood characterized by pervasive and impairing symptoms of inattention, hyperactivity and impulsivity of early onset and it causes significant functional impairment [1]. The worldwide pooled prevalence of this disorder was 5.29% [2], and many children continue to display significant symptoms throughout adolescence and adulthood [3]. The causes of attention deficit hyperactivity disorder were unknown. There were many etiological theories on the dysfunction of some brain areas that are implicated in inhibition of responses and functions of the brain [4-6]. Zinc plays an important role in the structure and function of the brain [7,8]. Iron and copper serve as cofactors in the synthesis of the brain neurotransmitters and magnesium is involved in the enzymes necessary for neurotransmitters release, as well as protecting neuronal cell membranes [9-11]. Changes in these minerals' levels may play a role in the pathogenesis and therefore the treatment of this disorder.

### Aim of the work

This study aimed to measure levels of zinc, ferritin, magnesium and copper in children with attention deficit hyperactivity disorder and compare them with normal healthy children.

## Methods

This study included 58 children aged 5-15 years with attention deficit hyperactivity disorder attending Minia University Hospital from June 2008 to January 2010 with a mean age 8.6 ± 1.8 years. They were classified into three sub-groups: sub-group I included 32 children with in-attentive type, sub-group II included 10 children with hyperactive type and sub-group III included 16 children with combined type according to the DSM-IV criteria of American Psychiatric Association, 2000 [1]. The control group included 25 apparently normal healthy children, 12 males (48%) and 13 females (52%) matched the study group in age, sex and socioeconomic state. Conner's Rating Scales were used in discriminating between children with ADHD and controls as well as severity of ADHD. Children with co-morbid neurological disorders, with chronic organic diseases as blindness or deafness, history of significant head trauma, history of perinatal asphyxia, significant hyperbilirubinemia or encephalitis and severely anemic children were excluded from this study and no medications were given at least one month prior to the study.

### Blood Samples

A blood sample of 2 mls were withdrawn by venipuncture after wrapping the skin by alcohol 70% and placed into a pyrogen-free tube for complete blood count. Another 2 mls of blood were withdrawn and centrifuged for serum separation and stored at - 70 degrees for other bioelements assay.

A colormetric test without desproteinization (SPINREACT) was used for zinc and copper assay. For ferritin assay a Direct ELISA Kit....The EiAsy TM Way was used. A colorimetric method was used for magnesium assay, and the coulter (Sysmex) system was used for hemoglobin assay [12-14].

All procedures were in accordance with the current revision of the Helsinki Declaration [15]. The parents or guardians of all children had to give informed consent to participate in the study. Patients whose parents or guardians refused to consent were excluded from the study.

### Statistical analysis

Values are given as means ± SD, range or as the number of subjects and proportions. The Student t test was used for group comparisons of normally distributed variables, and the Mann-Whitney U test and Wilcoxon signed-rank test were used for comparison of variables with skewed distribution. Pearson's test was used to calculate the correlation coefficient to describe associations between variables. One-way a nova test was used for analysis of biochemical variables. Both the independent sample t-test and chi-square tests were used to calculate p-value. P < 0.05 was considered significant. Analyses were performed using the SPSS software package (SPSS V 8.0 for Windows).

## Results

Patients and controls were comparable with respect to age, sex and residence. Paternal and/or maternal smoking and positive family history of attention deficit hyperactivity disorder were significantly higher in patients than controls *(p = 0.04 and p = 0.007 respectively)*(Table 1).

**Table 1:** Demographic data of the patients and controls

ITEM		Patients no = 58	Controls no = 25	p-value
**Age in years**	Range	5-13	5-15	0.6
	Mean ± SD	8.3 ± 1.8	8.6 ± 3.1	

**Sex**	Male	26(44.2%)	12(48%)	0.8
	Female	32(55.8%)	13(52%)	

**Residence**	Urban	29(50%)	17(68%)	0.1
	Rural	29(50%)	8(32%)	

**Smoking **(Paternal or maternal)	Positive	48(82.8%)	16(64%)	0.04*
	Negative	10(17.2%)	9(36%)	

**Family history of ADHD**	Positive	38(65.5%)	4(16%)	0.007**
	Negative	20(34.5%)	21(84%)	

* = significant

** = highly significant

Significant differences between patients and controls were present as regards to hemoglobin, zinc, ferritin and magnesium levels, with lower levels of them in patients than controls (*p = 0.04, p = 0.04, p = 0.03 and p = 0.02 respectively*). Copper levels were not significantly different *(p = 0.9) *(Table 2)

**Table 2:** Laboratory data of patients and controls

ITEM		Patients no = 58	Controls no = 25	p-value
**Hb (gm/dl)**	Range	9.3-13.3	9.9-14.7	0.04*
	Mean ± SD	11.8 ± 1.1	12.4 ± 0.9	

**Zinc (μg/dl)**.	Range	40-180	40-243	0.04*
	Mean ± SD	97.5 ± 29.4	117.4 ± 60.2	

**Ferritin (μg/dl)**.	Range	3-65	7-72	0.03*
	Mean ± SD	24.8 ± 14.1	32.6 ± 18.7	

**Mg (mEq/L)**.	Range	0.7-3.5	0.9-4.3	0.02*
	Mean ± SD	1.7 ± 0.8	2.2 ± 0.9	

**Copper (μg/dl)**.	Range	22-120	23-129	0.9
	Mean ± SD	45.4 ± 26.3	45.8 ± 23.05	

* = significant

**Figure 1:**
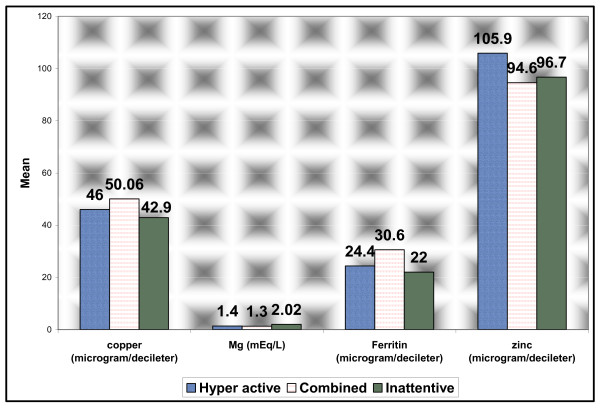
**Comparison between patients subgroups as regards laboratory data**.

There were no significant differences between patients subgroups as regards to hemoglobin, zinc, ferritin and copper levels (*p = 0.06, p = 0.5, p = 0.1 and p = 0.6 respectively*). Only magnesium levels were lower in both hyperactive and combined sub-groups than inattentive sub-group *(p = 0.01)*. (Figure 1)

**Figure 2:**
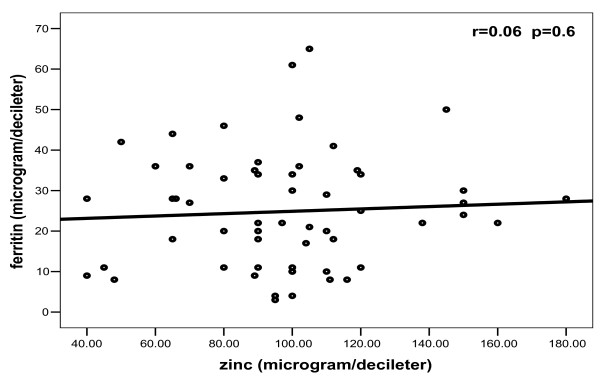
**Correlation between zinc and ferritin among patients**.

**Figure 3:**
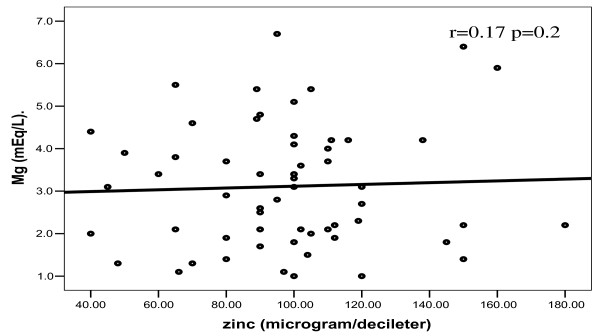
**Correlation between zinc and magnesium among patients**.

**Figure 4:**
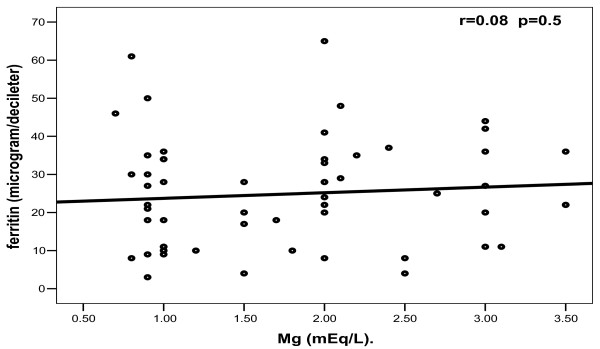
**Correlation between magnesium and ferritin among patients**.

**Figure 5:**
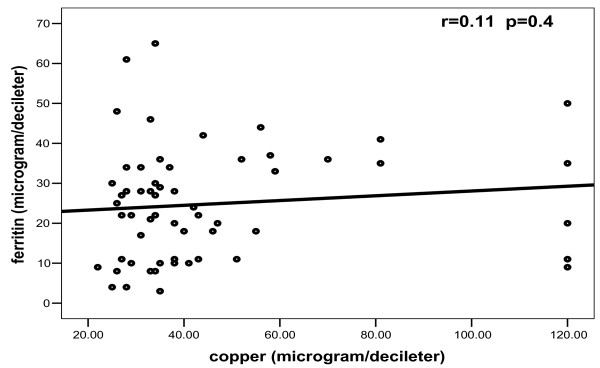
**Correlation between copper and ferritin among patients**.

In patients, no significant correlations were found between any one of bioelements studied and others (Figures 2, 3, 4, 5).

## Discussion

In this study, zinc levels were significantly lower in children with attention deficit hyperactivity disorder than controls. This was combined with significant lower levels of ferritin in in-attentive type, significantly lower levels of ferritin and magnesium in hyperactive type and significantly lower levels of magnesium in combined type.

Melatonin regulates dopamine function, which is widely believed to be a key factor in the pathogenesis of attention-deficit/hyperactivity disorder [16,17]. Higher Magnetic Resonance Imaging (MRI) of glutamate neurons observed in children with attention deficit hyperactivity disorder combined with a chelatable zinc pool in the synapses of these neurons may suggest higher zinc turnover in this disorder and possibly a higher zinc requirement in children with attention deficit hyperactivity disorder [18-22].

*Oner et al, 2010 *[23] reported that low zinc and ferritin levels were associated with higher hyperactivity symptoms in children. This is in accordance with our results which also in accordance with other studies reported that serum zinc levels correlate with parent and teacher rated inattention in children with attention deficit hyperactivity disorder [22-24].

Iron serves as a cofactor in the synthesis of important neurotransmitters such as dopamine, nor-epinephrine and serotonin [10] and deficiency in early years of life can negatively affect neural and behavioral development [25,26].

In this study, ferritin was significantly lower in children with in-attentive and hyperactive types than in controls; this is consistent with other studies [27,28]. No significant difference in ferritin levels was found between children with combined type and controls, and this is not in accordance with the previous studies. This may be due to relatively higher level of hemoglobin in children with combined type in our study with no significant difference as regards hemoglobin level between children with combined type and controls.

In agreement with other studies [29,30], magnesium levels were significantly lower in both children with hyperactive and combined types than in controls and this may be due to the role of magnesium in protecting cell membranes from excitatory neurotransmitters such as glutamate.

*Riley et al, 2008 *[31] reported that preschool children with hyperactive and combined types of attention deficit hyperactivity disorder demonstrated similar levels of functioning and they suggested that hyperactive type may represent an earlier form of combined type. This supports our results as both children with hyperactive and combined types were lower in zinc and magnesium levels than controls.

*Bosc et al, 2004 *[32]*and Mousain et al, 2006 *[33] reported that magnesium/vit.B6 intake reduces central nervous system hyper-excitability in children with attention deficit hyperactivity disorder and this supports our results as magnesium levels were significantly lower in both hyperactive and combined types but not in in-attentive type.

Copper is an essential factor for both the development and function of the central nervous system. It acts as a cofactor for several key enzyme systems, most notably dopamine hydroxylase [34]. In this study, there was no significant difference between children with attention deficit hyperactivity disorder and controls as regards copper levels and this is not in accordance with other studies which had reported lower levels of copper in those children [12], nor in accordance with other studies reported that excess copper may cause hyperactivity, mood swings, anxiety and anti-social behavior [34].

A history of smoking, either paternal or maternal, was significantly higher in children with attention deficit hyperactivity disorder than in controls. This is in accordance with many studies reported that maternal smoking during pregnancy is a risk factor for many cognitive and behavioral disorders [35,36].

A positive family history of attention deficit hyperactivity disorder was also significantly higher in patients than controls and this is in accordance with other studies which had reported that attention deficit hyperactivity disorder shares familial and genetic factors [37,38]. *Smidts et al, 200*7 [39] reported more prevalence of attention deficit hyperactivity disorder in boys than girls while *Dong et al, 2008 *[40] reported the reverse. This study showed no significant difference between girls and boys or between rural and urban children. This may be attributed to the selection of our controls whose age, sex and socioeconomic state were matched with patients. Limitations of this study included the small sample of patients - only 58 children - but this may be attributed to Egyptian culture which consider regular visits of neuropsychiatry unit is shameful as well as low incidence of attention deficit hyperactivity disorder in general.

## Conclusions

Children with attention deficit hyperactivity disorder had lower levels of zinc, ferritin and magnesium than healthy controls but had normal copper levels. Zinc levels were significantly lower in all types of attention deficit hyperactivity disorder while ferritin levels were significantly lower in both in-attentive and hyperactive types. Magnesium levels were significantly lower in both hyperactive and combined types than in control group. These results may be beneficial in treatment of children with attention deficit hyperactivity disorder according to their types.

## Competing interests

The authors declare that they have no competing interests.

## Authors' contributions

All authors read and approved the final manuscript. MMM conceived and designed the study and revised the manuscript for important intellectual content. He will act as guarantor of the study. AME was responsible for history taking and clinical examination of children of this study. He analyzed the data and helped in manuscript writing, revision and submission. RMM helped in clinical examination of children and selection of cases of the study as well as final revision of the manuscript. MMS conducted the laboratory tests and interpreted them.
